# From science to society: implementing effective strategies to improve wild pollinator health

**DOI:** 10.1098/rstb.2021.0165

**Published:** 2022-06-20

**Authors:** Jane C. Stout, Lynn V. Dicks

**Affiliations:** ^1^ School of Natural Sciences, Trinity College Dublin, College Green, Dublin 2, Ireland; ^2^ Department of Zoology, University of Cambridge, Downing Street, Cambridge CB2 3EJ, UK

**Keywords:** conservation, ecosystem services, IPBES conceptual framework, multiple benefits, pollinator decline, pollinator policy

## Abstract

Despite a substantial increase in scientific, public and political interest in pollinator health and many practical conservation efforts, incorporating initiatives across a range of scales and sectors, pollinator health continues to decline. We review existing pollinator conservation initiatives and define their common structural elements. We argue that implementing effective action for pollinators requires further scientific understanding in six key areas: (i) status and trends of pollinator populations; (ii) direct and indirect drivers of decline, including their interactions; (iii) risks and co-benefits of pollinator conservation actions for ecosystems; (iv) benefits of pollinator conservation for society; (v) the effectiveness of context-specific, tailored, actionable solutions; and (vi) integrated frameworks that explicitly link benefits and values with actions to reverse declines. We propose use of the Intergovernmental Science-Policy Platform on Biodiversity and Ecosystem Services (IPBES) conceptual framework to link issues and identify critical gaps in both understanding and action for pollinators. This approach reveals the centrality of addressing the recognized *indirect* drivers of decline, such as patterns of global trade and demography, which are frequently overlooked in current pollinator conservation efforts. Finally, we discuss how existing and new approaches in research can support efforts to move beyond these shortcomings in pollinator conservation initiatives.

This article is part of the theme issue ‘Natural processes influencing pollinator health: from chemistry to landscapes’.

## 1. Introduction

A huge variety of animals act as pollinators to the vast majority of plant species on Earth [1], and thus play a fundamental role across natural and managed ecosystems. Despite this, the health of many pollinating animals (at individual, population and community level, across a range of taxa, both managed and wild) is thought to be declining worldwide [2]. Pollinators play a fundamental role in many terrestrial ecosystems and benefit the production of 75% of the most common human food crop species [3]. Consequently, much of the concern about pollinator decline has focused on the implications for crop production [4,5]. While this is important for healthy diets, agricultural economies and nutritional security [6], nearly 90% of the world's flowering plant species are animal-pollinated [7]. An estimated one-third of all wild plant species would produce no seeds at all, and half would experience an 80% reduction in fertility, in the absence of pollinators [8]. Thus a decline in the health of pollinating animals can have far-reaching impacts on human health and well-being, economy and society, within and beyond the agricultural sector, and damage the structures and processes of ecosystems, as well as their ability to deliver other ecosystem services [9,10].

Decline in pollinating animals has become a popular and prominent example of how biodiversity loss can affect humanity, in both public and private discourses, usually with reference to the potential impacts of decline on food production. While in the 1960s and 1970s, scientific attention focused largely on risks associated with agriculture and managed bees (particularly honeybees), by the 1990s, the wider importance of plant–pollinator interactions was acknowledged in the literature [11]. Since the ‘Earth Summit’ in Rio de Janeiro, Brazil, in 1992, which was a turning point in recognition of the value of biodiversity to society, both scientific research into pollinators and pollination, and political interest, have increased [12]. In 2002, the United Nations Convention on Biological Diversity adopted the ‘International Pollinator Initiative for the Conservation and Sustainable Use of Pollinators' (IPI) [13]. Subsequently, widely publicized issues, including colony collapse disorder in North American honeybees (*Apis mellifera*) in the late 2000s [14], the impacts of neonicotinoid pesticides in the early 2010s [15], and the first thematic global assessment conducted by the Intergovernmental Science-Policy Platform on Biodiversity and Ecosystem Services (IPBES) on ‘Pollinators, pollination and food production’, published in 2016 [6], all raised awareness of the decline and importance of pollinating animals in the collective public and political consciousness.

**Figure 1.**
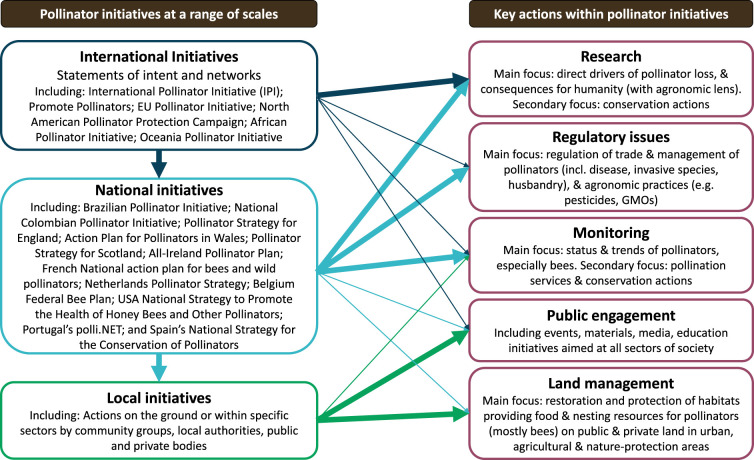
Summary of current pollinator initiatives and the main actions they promote for pollinator conservation. Initiatives at different scales have greater (thick arrows) or lesser (thin arrows) impacts on actions taken. (Online version in colour.)

This has resulted in numerous public and private initiatives and policies, and widespread enthusiasm for the protection and conservation of pollinators, particularly bees [16], including agri-environment schemes and pesticide restrictions, urban and commercial initiatives and ‘rewilding’ at various scales. Initiatives range from global (e.g. the IPI, and the Coalition of the Willing on Pollinators—Promote Pollinators), to continental (e.g. EU, North America, Africa, Oceania), to national and local scales (figure 1).

International initiatives tend to encompass broad-scale statements of intent and facilitate networks for promoting awareness and knowledge-sharing. For example, the original IPI aimed to ‘promote coordinated worldwide action to monitor pollinator decline’, improve management of pollination services for ‘sustainable agriculture, and enhance food security, nutrition and livelihoods through conservation, restoration and sustainable use of pollinators' [17]. The IPI's website provided case studies, technical documents and tools (including for species identification). The IPI was updated for the period 2018–2030, and emphasized the need to mainstream pollinator and pollination issues in policy, develop and implement actions to support pollinator conservation, address risks to pollinators, build capacity and knowledge-sharing for how to integrate pollinator issues into land-use decisions, and promote collaborative research. Although facilitated and coordinated by the Food and Agriculture Organization of the United Nations (FAO's Global Action on Pollination Services for Sustainable Agriculture), the IPI is not a funded entity and the FAO has so far had little capacity to implement its objectives. The ‘Coalition of the Willing on Pollinators' (Promote Pollinators) was initiated in 2016 during the Convention on Biological Diversity's Conference of the Parties (CBD COP13) and is a growing alliance of (currently 30) ‘countries and observers who believe that country-led politics can foster policy measures and innovative action to protect pollinators' [18]. Its secretariat is financed by the country that holds the presidency and has thus far been financed by the Ministry of Agriculture, Nature and Food Quality of The Netherlands. The organization releases statements agreed by members, coordinates and runs events and publishes a newsletter.

At continental scale, the North American Pollinator Protection Campaign [19] aims to promote pollinator health across Canada, the USA and Mexico, and has organized annual conferences (since 1997), creates task forces to deliver specific objectives, and is developing a strategic plan for conservation across North America. By contrast, the EU Pollinators Initiative [20] has set long-term objectives (to 2030), as well as actions under three priority areas: (1) improving knowledge of pollinator decline, its causes and consequences; (2) tackling the causes of pollinator decline; and (3) raising awareness, engaging society-at-large and promoting collaboration. It was recognized that improved communication and coordinated national strategies and action across EU member states would be beneficial to inspire, promote best practice, and address potential conflicts to ultimately address pollinator declines [21]. Other continental initiatives (e.g. the African and Oceania Pollinator Initiatives) are specific in their goals, appear to be less active at the moment, and specifically highlight pollinator conservation to support pollination services for agriculture.

Most of the national-level strategies focus on education/awareness raising, habitat creation/land management, improving the knowledge base/research, supporting managed pollinators and beekeepers and tracking change over time. Some specifically focus on reducing risks from pests and disease (e.g. Pollinator Strategy for England), invasive species and pesticides (e.g. Spain's National Strategy), and on determining the value of pollinators, mainstreaming and capacity-building (e.g. National Colombian Pollinator Initiative). National-level ambitions range from broad, high-level statements of intent to providing direct support for sectoral-specific actions (e.g. the All-Ireland Pollinator Plan), and some are associated with dedicated government funding [21].

Despite increasing activity across sectors and scales, widespread reversal of pollinator decline has not yet been achieved. Pollinators are still declining, albeit possibly at a slower pace than previously in some countries [22]. In this paper, we briefly review the state of knowledge for implementing effective pollinator conservation, propose a framework for a holistic approach and identify where pollinator strategies should focus efforts going forward.

## 2. Implementing effective pollinator conservation actions

While overarching strategies and plans can promote and coordinate efforts, it is usually up to individual countries, regions within countries and individual land-owners/managers to implement conservation actions on the ground (figure 1). It is beyond the scope of this paper to analyse all these initiatives in detail, but other authors have highlighted inadequacies in pollinator conservation policies at various levels. For example, despite an increasing number of subnational insect pollinator policies (passed by state-level legislatures) coming into existence and showing potential in the USA, these policies lack several components identified by scientists as necessary to tackle the pollinator decline crisis, including shifting to more diversified farming systems and reducing dependence on insecticides [23]. Furthermore, while public awareness of pollinator decline and the need for conservation has improved, certainly in the EU and in the USA [23], fundamental misunderstandings persist, especially around the role of honeybees and beekeeping in pollinator conservation [16,24]. There is also a policy implementation gap, partly stemming from insufficient understanding of how to effectively enable behaviour change [25]. Many initiatives rely on voluntary action, but there is not enough understanding of what motivates people to volunteer [26]. Failure to implement strategic coordinated conservation actions for pollinators can also be due to financial or budgetary constraints, research silos, limited availability of appropriate expertise, political focus on promoting honeybees or lack of political will to support changes to industry (especially agriculture), and lack of ‘champions’ or individuals willing and able to work across disciplines, sectors and scales [27,28].

These examples serve to illustrate the importance of compiling relevant knowledge (including both scientific, and indigenous and local knowledge) at an appropriate scale, before designing effective actions for the reversal of pollinator decline. A large proportion of recent research on pollinator conservation has focused on how direct drivers of pollinator decline influence pollinator abundance and diversity, and the implications for pollination services to human food crops [6]. In reality, this has resulted in an ever-expanding knowledge base on generalist pollinators, especially European honeybees, *Apis mellifera,* and a couple of bumblebee species (mainly *Bombus terrestris* and *Bombus impatiens*), but most of the diversity of pollinators, and of plant–pollinator interactions on this planet, are still poorly understood [12,29,30]. Thus, the knowledge currently being generated by the research community, while relevant, does not provide the full range of knowledge needed to inform effective pollinator conservation to improve pollinator health in its broadest sense. In the following sections, we define the knowledge domains that will be critical in designing effective pollinator conservation actions, provide an overview of knowledge in each domain and describe where there are gaps in current understanding.

### (a) Status and trends of pollinators

Reasonably good distribution data are available at global scale for the vertebrate pollinating taxa, especially birds and mammals [31]. However, there are substantial knowledge gaps with regards to the current status and trends for most invertebrate pollinators across much of the world [32]. Even in Europe, where there has been considerable research and recording, the conservation status of 57% of bee species has not been assessed owing to lack of data [33]. Obtaining good invertebrate pollinator distribution data requires substantial investment of time, money and taxonomic expertise [34], and is rarely employed at the temporal and spatial scale needed, despite the economic arguments for it [35]. Modelling species distributions, when there is sufficient knowledge of pollinator biology, landscapes and land-use change, climatic conditions and other factors, is growing as a tool to address these data gaps, both at national scale [36] and for particular species (e.g. for bumblebees [37]). It is essential to know which pollinators are at risk, and in which places, so that conservation actions are targeted at the species and in the places where they are most needed.

### (b) Drivers of decline

Wild pollinator decline is caused by a combination of indirect drivers, such as human population growth, urbanization, technology and globalization of trade, and direct drivers, such as habitat loss, disease and exposure to toxins [38]. At large continental and global scales, there is reasonable consensus that the most important direct drivers of pollinator decline are changes in land cover and configuration (i.e. usually involving habitat loss), land management and pesticides [10,39]. All of these can be strongly linked to processes of agricultural intensification and urbanization, which are themselves a result of demographic, economic, political and institutional indirect drivers, underpinned by societal values.

Recent and on-going research focuses on combinations of direct drivers of decline, since drivers never act in isolation (e.g. Brown *et al*. [40] are examining the role of agrochemicals, parasites and disease, and nutritional stressors on managed species of bees). The complexity involved with understanding the impacts of multiple direct drivers of decline across individuals, populations and communities has necessitated empirical studies at a range of scales (field, semi-field and laboratory), integrated with risk-assessment and ecological modelling. The huge range of species (and our lack of knowledge about most of them), environmental contexts and interactions of drivers (different types of agrochemicals, diseases, nutritional stressors and their interactions) mean that it is not possible to conduct field, semi-field or laboratory tests for all possible situations, or even to model every scenario. This means that our knowledge of the impacts of direct drivers, and how they interact in real-world contexts, is extremely limited.

Understanding the indirect drivers demands even more complex, multidisciplinary research [41], while addressing them requires transformative change of economic, political and social institutions and governance [41–43]. Quite understandably, these issues are usually considered out of scope for conservation strategies focused on pollinators, even at large scales.

### (c) Multiple benefits of pollinator conservation for ecosystems

Pollinator conservation can have multiple positive impacts on ecosystems, including by (i) increasing abundance and diversity of target species of pollinating animals, (ii) increasing biodiversity of other taxa and habitats, and (iii) benefitting ecosystem processes. There are potential negative implications of conservation efforts as well, for example, if only certain species, locations or actions are focused on, at the expense of others. Indeed, much of the habitat restoration for pollinators has been implemented on agricultural land, through agri-environmental schemes (AES) and other initiatives, primarily targeting a small range of generalist bee species that play a role in crop pollination, and benefitting those that can persist in these highly modified landscapes, rather than the wide array of bee species [44–47]. In addition, these efforts usually focus on providing more nectar and pollen in a landscape, but non-bee pollinating animals need a diversity of food throughout the season and for different life stages, and all pollinators also need non-food resources, such as nesting, egg-laying or overwintering sites [48]. There is relatively little detailed knowledge of what most species require [49] and there has been little long-term monitoring of the impact of such actions on pollinator population growth rates or persistence. While there is some evidence that AES can enhance pollinator abundance and survival, particularly in arable landscapes of moderate complexity [50,51], AES need to be properly designed and targeted [52], and efforts at a large scale have had limited success [53,54].

Urban habitats can also support a wide range of pollinator species [55], presenting opportunities for conservation actions in towns and cities, which are frequently a focus of local pollinator initiatives (figure 1). As in agricultural landscapes, the focus of conservation effort in urban areas has generally been on providing floral resources [56] and we do not know of any long-term monitoring of the effectiveness of actions.

Beyond the target species, pollinator conservation actions could potentially benefit a wide range of taxa, from all trophic groups, depending on the type and scale of the action. For pollination-limited plant species currently facing pollination deficits, increasing pollinator density can increase fruit and seed production. This has been demonstrated to be the case at global scales through meta-analysis for crops across many agricultural systems [57] and for wild plants, especially in areas of high species richness [58]. Facilitating increased fitness of animal-pollinated plants, and intentionally providing floral resources for pollinators, can benefit other herbivorous animals, including those that consume the fruits and seeds [59]. Increasing insect pollinator numbers could, therefore, benefit wild birds and other insectivorous animals and natural enemies (but see [60]). Conversely, some actions, such as reduced mowing, and encouraging longer swards, may have negative impacts on other taxa, e.g. birds that hunt/forage on close-cropped lawns [61].

Plant taxa that benefit from enhanced pollination may themselves play important ecological roles, through which pollinator conservation can potentially enhance important ecological processes. For example, actions that increase plant biomass may contribute to increased primary production overall, to nutrient cycling (e.g. nitrogen fixation), to water retention or to regulating climates. It has long been determined that diverse plant communities can have greater ecological stability [62,63], which in turn can provide resistance to climate events [64]. In addition, pollinators contribute to the production of woody reproductive litter (e.g. woody seed casings and dispersal structures), which enter the soil carbon store [65]. While synergies have been identified between actions for climate and biodiversity, for example, there is a positive relationship between soil organic carbon stocks and invertebrate diversity [66], the potential knock-on benefits of pollinator conservation actions for other taxa and processes, and in which biogeographical contexts, have not been empirically determined.

### (d) Multiple benefits of pollinator conservation for society

There are considerable knowledge gaps regarding the impacts of pollinator decline and implementation of conservation actions on human society and economy. While there is a growing understanding of how to assess the multiple values of nature to encompass a range of world views, knowledge and value systems [67], there is a gap in methodological approaches that directly focus on pollinator impacts on human well-being, especially from a socio-cultural viewpoint [10]. The value of pollinators is almost always quoted in terms of contribution to human crop production [68], and non-market values and co-benefits are not well articulated or quantified [69].

Some pollinators, especially non-bees, perform other ecological functions and provide additional services, and so pollinator conservation measures can have other benefits, particularly in agricultural systems. Many hoverfly species (Diptera: Syrphidae), for example, provide economically valuable pest regulation through aphidophagy in their larval stages, while other groups of pollinating flies are detritivores (e.g. Syrphidae: Eristalini) or scavengers and decomposers (Diptera: Calliphoridae). Effective actions that benefit them in their pollinating adult stage could clearly have potential knock-on benefits for these services delivered by the larval stages [70]. Aculeate wasps, as well as acting as pollinators, play significant roles as predators, decomposers and seed dispersers [71]. Other taxa that may benefit from pollinator conservation include plants that play an important role in landscapes in terms of microclimate regulation and soil erosion prevention, and as windbreaks [70]. These potential wider co-benefits of pollinator conservation for ecosystem service delivery have not been well researched.

The range of benefits that arise from the conservation of wild pollinators, pollination and other ecosystem processes, for both nature and people, is considerable. These benefits, and the full extent of repercussions for human society and economy of declines in pollinator health, and conservation actions to reverse these declines, could be articulated using the natural capital approach. This is a holistic approach, whereby human impacts and dependencies on natural systems are explicitly identified and accounted for in order to improve policy and decision-making [72,73]. International and national policies have embraced this approach and pledged to incorporate natural capital into national accounting (including Sustainable Development Goal 15.9, the EU Biodiversity Strategy). To do this, several natural capital accounting and assessment frameworks have been developed, including the UN System of Environmental Economic Accounting-Ecosystem Accounting (SEEA-EA), mostly used by public bodies at national or regional scale [74,75], and corporate natural capital accounting approaches, used at organization scale [76]. Spatially explicit SEEA-EA for pollination has been generated using maps of potential service provision by pollinators, based on wild and managed bee distribution data at national scales [77], and, for a limited number of species, distribution records at international scales [78], as a proxy for pollination supply. Demand for pollination service is usually represented by the extent of pollinator-dependent crops [79], and, if multiplied by the pollinator dependency of each crop, can highlight areas of higher pollinator demand at regional scales [80]. Using this approach, the overlap between supply and demand is used to estimate service flow, which is then integrated into accounts. More nuanced models of pollination service flow have been developed, including the Natural Capital Project's InVEST Crop Pollination Model [81]. In this case, the models predict relative abundance of pollinators based on maps of pollinator nesting and floral resources in the landscape, and on foraging distances of pollinators. These can then be integrated into accounts. However, these approaches need good baseline data on distributions of pollinators and habitats, as well as pollinator resource requirements and behaviour, which are lacking for most taxa in most parts of the world. In addition, attempts to incorporate pollination into accounts have so far been limited to crop pollination, and demand for pollination service for wild plants has not been modelled, nor have any of the wider societal benefits from pollinators.

### (e) Tailored and actionable solutions

Public, political and scientific interest in pollinator conservation has popularized the implementation of conservation actions for pollinators across the world [82]. Indeed, there is great potential for conservation actions to be implemented in a range of sites, including urban [83]. Although an abundance of online material promotes bee conservation, better understanding of how some actions actually contribute to pollinator conservation is required [84]. For example, the role of beekeeping in pollinator conservation is often misunderstood [24,85]. Although some general actions for insect conservation have been articulated [86], context-specific, tailored and actionable solutions are relatively rare.

Provision of habitat and resources tends to focus on a small proportion of bee species, and rarely focuses on provision of non-floral resources [48], and there is often little or no assessment of how successful conservation actions are (but see [87]), or how frequently they are implemented across landscapes [26]. In addition, increased awareness and enthusiasm among wide swathes of society can result in well-meaning actions for pollinators employed at local levels (e.g. planting flowers, installing bee hotels, taking up beekeeping) that are not always based on scientific understanding, and can be ineffective or even harmful to wild pollinators if implemented or located wrongly.

To reverse pollinator loss, drivers of decline need to be removed, habitats must be restored, and the ecological, economic and societal business cases for action need to be articulated. There is a need for effective changes in policy and behaviour across all sectors, and at a range of governance levels, from individual land-managers, local communities and small and medium enterprises, to local, national and regional governments, and multinational businesses [88]. To reverse wild pollinator decline, key stakeholders from policy makers to business owners and the wider society need to be given a voice. Although this has begun in some places, and there is evidence that co-production can bring multiple gains, there is still a lot of progress to be made [23,89].

### (f) Integrated frameworks and tools

For informed policy-making, features of society and economy need to be linked with the biosphere, allowing consideration of interrelations and interdependencies between conservation actions, drivers and impacts, and their relative importance [90]. We can make predictions about how drivers of decline and positive pollinator conservation action will make an impact on pollinators, and what the impacts of decline/conservation actions may be on social, environmental and economic systems, but an integrated framework is needed for meaningful decision-making. Integrated assessment models (IAMs) can improve our understanding of these dynamic systems, inform decision-making on implementation of conservation actions, facilitate integration of pollinators and pollination into life cycle analysis [91], and improve accuracy of Earth system models more broadly [92]. IAMs have been used to inform policy-making, usually in the context of climate change [93], but they have not been so widely used in forecasting scenarios associated with habitat change [92]. IAMs are based on input scenarios, developed in consultation with stakeholders [94], forecasting what could happen in the future. IAMs then offer projections on the plausible changes that could occur over a fixed time scale. This could aid understanding of the drivers and impacts of pollinator decline and conservation actions, both for pollinators, and for other taxa, ecosystems and service provision.

The most popular types of IAMs are multi criteria decision analysis (MCDA) and Bayesian belief networks (BBN), which allow integration of perspectives from different disciplines and stakeholders, incorporating values and uncertainties [90,95]. The IPBES global assessment [6] brought together evidence on values, status and trends of pollinators, drivers, risks and responses to pollinator decline. This was used as the basis for a ‘drivers, risks and responses' framework by Potts *et al*. [2], but the strength of the connection between elements in the framework was based on the clarity of evidence, not the strength of the relationship.

One of the challenges in developing integrated frameworks is collecting and bringing together data that are logistically difficult or expensive to obtain from experiments, although this can be overcome using structured expert elicitation (e.g. [96]). However, there is clearly scope for further application of integrated frameworks to tackle the complexity of restoring global pollinator health.

## 3. Framing the issues

To address and reverse wild pollinator decline, effective and integrated changes in policy and behaviour are needed, across all sectors, not just within agricultural or nature-orientated spheres. To enable this, a utilitarian approach is usually taken, explicitly linking the benefits and values of pollinators to human well-being with actions to reverse their decline (e.g. [6]). This approach can raise awareness of the issue of pollinator decline, the risks associated with not taking action, and the potential actions for conservation, and is often aimed at interdisciplinary researchers, non-scientists and decision-makers. One framework that has been developed to explicitly link people and nature for such audiences is the Intergovernmental Science-Policy Platform on Biodiversity and Ecosystem Services (IPBES) conceptual framework [97]. This recognizes several components of natural and social systems, operating at various spatio-temporal scales, and was specifically developed to address the fact that reversing biodiversity loss requires interdisciplinary collaboration, and that institutions, governance and decision-making play a central role [97]. Despite receiving criticism for being centred on economic norms and instrumental values [98], the framework was adopted to communicate the core concepts of the IPBES Assessment Report on Pollinators, Pollination and Food Production Summary for Policymakers [99]. In this way, the conceptual framework explicitly addressed issues regarding pollinators and linked them under three headings: A. Values, B. Drivers and management options, and C. Status and trends. The framework has also been applied in an attempt to improve policy effectiveness in particular ecosystem types, for example to guide policy for the conservation of South American grasslands [100], and to test whether research is addressing all the issues for the conservation of the Southern Cape flora [101].

Potts *et al*. [2] built on this, and linked the major drivers of pollinator decline with risks to human well-being, alongside societal responses to address drivers (including knowledge, action, infrastructure, technology, organizations and governance). This enabled assessment of current understanding of the strength of knowledge of links between responses and drivers, and drivers and risks, and identification of gaps in knowledge, and concluded that more research on drivers and driver interactions was required to make responses more targeted [2]. Following this, Dicks *et al*. [10] examined the perceived relative importance of drivers and risks of pollinator loss. This revealed considerable scientific uncertainty about the implications of pollinator loss for society, and major geographical knowledge gaps [10].

Here we propose further application of the IPBES conceptual framework specifically to the pollinator decline and conservation issue, to develop understanding of the full implications of pollinator decline and humanity's responses to address it, not just for human well-being, but for natural and managed ecosystems more broadly. We argue this framework can be used to bring together the interlinked issues to develop better understanding of what we are currently missing, and where the critical action gaps are.

**Figure 2.**
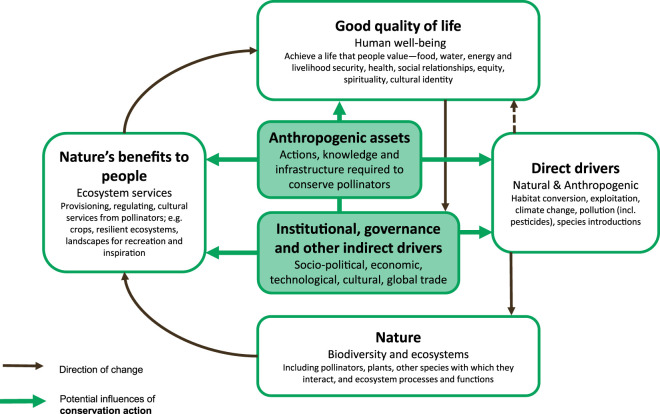
Conceptual framework for integrated assessment of drivers of pollinator decline and the impacts on nature, nature's benefits to people, and human well-being (adapted from Diaz *et al*. [97]). (Online version in colour.)

Figure 2 shows the IPBES conceptual framework for integrated assessment of drivers of pollinator decline and the impacts on nature, nature's benefits to people, and human well-being (adapted from Diaz *et al*. [97]). The key elements of the framework are:

(1) **Direct drivers**—factors that directly affect pollinators include natural climate and weather patterns, which are beyond human control, and drivers that are the result of human behaviour (anthropogenic drivers), which can exacerbate naturally occurring processes. Conversion and exploitation of natural habitats, climate change, species introduction and pollution (including by pesticides) are the direct drivers of wild pollinator decline and other biodiversity loss, as well as pollination and other ecosystem functions. They are the result of indirect drivers and affect the state of nature.
(2) **Nature**—encompasses the biosphere, atmosphere, geosphere and hydrosphere, which make up our global stock of natural capital. The biosphere includes pollinators, plants, other species with which they interact, and ecological processes and functions. The status and trends of ‘nature’ are influenced by the direct drivers.
(3) **Nature's benefits to people**—these are the ecosystem services, or benefits, that flow from nature to people. The benefits from wild pollinators include provisioning, regulating and cultural ecosystem services (biomass production, pollination, resilience to change, landscapes for recreation and inspiration). These benefit society and increase quality of life, and their provision is influenced by the state of nature, indirect drivers of loss and anthropogenic assets.
(4) **Good quality of life**—refers to human well-being and society, and people being able to achieve a life they value, with food, water, energy and livelihood security, health, social relationships, equity, spirituality and cultural identity. The quality of life influences societal institutions and governance.
(5) **Anthropogenic assets**—these include infrastructure, knowledge, technology and financial assets (human, manufactured and financial capital) that support conservation actions for wild pollinators. They will be key to influencing drivers of decline, wild pollinator status and trends to reverse decline, benefits to people and quality of life. The capacity for society to engage in these actions depends on the quality of life, good governance and institutional support, as well as access to opportunities, evidence-based good practices, and training.
(6) **Institutional, governance and other indirect drivers**—indirect drivers of pollinator decline include economic development, trade and finance, technological developments and demographic trends. These are influenced by the way society organizes itself in terms of institutions and governance, and are the underlying causes of change. These indirect drivers play a key role in influencing how people and pollinators interact, wild pollinator status and the benefits that people derive from nature.

This framework is useful for designing effective pollinator conservation strategies and actions because it is more specific than any that have been applied previously. For example, the ‘Drivers, Risks and Responses' framework by Potts *et al*. [2] highlights that responses are the result of anthropogenic assets and institutions and governance, but fails to separate out these elements. Nor is there a link in their framework between quality of life and well-being, and the responses.

The IPBES framework (figure 2) highlights the central role of society in addressing pollinator decline and emphasizes that assets and institutions are fundamentally different but linked. It is no accident that these elements sit in the centre of the framework. Framing conservation activity as ‘anthropogenic assets’, supported by institutions and governance, makes clear the core role of people in pollinator conservation and underlines the importance of engaging a wide range of stakeholders in the design process. It is also important to note that the only incoming influence on the indirect drivers of change in the framework comes from the ‘Good quality of life’ box. This indicates that transformative, societal change, as opposed to individual behaviour change, happens when large numbers or a majority of people recognize that their quality of life, livelihoods and security are threatened or degrading in the current context. Thus, to reverse drivers of decline, pollinator conservation strategies must take action on institutional indirect drivers of decline. Almost none of the strategic efforts that are currently in place do this explicitly.

## 4. Where now for pollinator conservation?

In this paper, we have considered the hierarchical structure and content of existing global efforts to reverse pollinator declines; defined and reviewed the knowledge domains we consider critical to designing effective pollinator conservation actions; and used the IPBES conceptual framework to discuss how pollinator conservation might broaden its scope to enable it to drive the transformative change that many others have argued will be necessary to reverse biodiversity loss [42].

In this final section, we consider how each of the areas of action frequently included in pollinator initiatives (figure 1) could be enhanced or improved, based on the critical knowledge domains and gaps we have identified, and the conceptual framework we propose.

### (a) Research

Research gaps that are crucial to implementing effective pollinator conservation actions have been outlined above, but the key issue is that the concept of pollinator-relevant research needs to be broadened. To date, much of the research attention has focused on status and trends of pollinators (the ‘Nature’ box in our conceptual framework (figure 2)), and on direct drivers of decline. While these areas still warrant study, the wider benefits for ecosystems, co-benefits for nature's contributions to people, and the effects on human quality of life and well-being all face significant knowledge gaps. Furthermore, the linkages between these domains, alignment of pollinator conservation actions with other environmental challenges, strategies to effect behavioural change at societal scale, as well as indirect institutional drivers of change, have not been prioritized in research to date. This requires pollinator research to encompass multiple disciplines and not remain the sole remit of ecologists. Although this has started to happen, particularly with the increased popularity of pollinators to funding bodies, meaningful collaboration across disciplines, and across geographical regions, should be prioritized and encouraged.

### (b) Regulatory issues

This area of pollinator initiatives falls in the centre of our proposed conceptual framework, as it has potential to connect directly into institutions and governance. However, regulatory issues in existing initiatives (figure 1) generally focus on trade and husbandry of managed pollinators (particularly the honeybee *Apis mellifera*) and their associated diseases, along with regulations or policy related to agricultural practices, such as agri-environmental schemes and pesticides. While these areas are clearly important to protect pollinators, they tend to be relatively narrow in focus and somewhat ‘end-of-pipe’, in that they address the direct drivers of change, rather than the indirect drivers that underlie them.

As an example, a radical step to support pollinators was taken by the EU, when it banned some neonicotinoid insecticides from outdoor agricultural use in response to evidence of adverse impacts on wild bee populations. The benefit to pollinators may be temporary, however, because new types of systemic insecticide are coming to market to replace neonicotinoids, which have the same mode of action and similar sub-lethal effects on bees and predatory arthropods [102]. By not addressing underlying indirect drivers of pollinator decline (the processes of global trade, human population growth, dietary change and unsustainable development which drive agricultural intensification), the action risks being ineffective in the long term.

Agri-environment schemes targeted at pollinators often focus on providing a limited range of nectar and pollen resources through simple management prescriptions for farmers. These provide resources for common, generalist species of pollinator, rather than supporting the more specialist pollinator species that are actually declining [46,103,104], and seldom provide resources at times in the year when resources are actually limiting [105]. These actions build some anthropogenic assets (knowledge and infrastructure to increase floral resources in the landscape), and partially address the land use/habitat loss-related direct drivers of decline, but they require public subsidy that may not be sustainable in the long term and, if non-native species and/or ecotypes are used, may degrade biodiversity.

To broaden and deepen the impact of regulatory actions in pollinator initiatives, we suggest that architects of national initiatives consider the potential for regulations that address the indirect drivers of pollinator decline. Regulatory environments that demand full natural capital accounting are one promising area (see above). Incentives, policies or regulations that focus on overall system transformation can be envisaged, such as the French Écophyto target to reduce overall dependence on pesticide inputs in agriculture [106], or requirements for life cycle impacts of products traded from overseas to be communicated in product labelling.

### (c) Monitoring

Long-term, standardized and systematic monitoring of pollinators is required at large scales if we are to have a clear picture of what is happening to pollinators globally and understand whether conservation efforts are having the desired effect. In the EU, there are proposals for a pollinator monitoring scheme [107], incorporating citizen science approaches, but this needs testing and validating, and monitoring needs to extend beyond Europe. Citizen science schemes exist in many countries around the world (for example, there are citizen science schemes focused on pollinators in Brazil, Australia, North America and India, among others). Effort is required to capture and integrate the data these schemes collect, both nationally and globally. Taxonomy is a challenge for monitoring pollinators, especially in tropical regions, but there is ample experience of establishing recording schemes that target easy-to-identify species. The capacity to establish online biodiversity observation systems that meet international standards and link to global databases is increasing all the time [108]. In addition, models to predict and make accessible the occurrence of the main wild pollinator species and groups at the landscape scale are required.

### (d) Public engagement

The IPBES conceptual framework (figure 2) helps to identify the links, from degrading ecosystems (Nature), through declining ecosystem services (Nature's benefits to people), to people's quality of life, that need urgently to be better understood and communicated in pollinator conservation efforts. Similarly, the connection between institutions and governance and direct drivers of pollinator decline, shown clearly in the framework, could be more transparently explained. Large-scale societal trends such as global trade and demography can feel far removed from individual people and beyond our power to change things, and as noted above, these elements are seldom mentioned in pollinator conservation strategies and documents. It would be possible, for example in national strategy documents, or educational material, to acknowledge the ‘virtual biotic pollination flows' recently quantified by Silva *et al*. [109], through which demand for pollinator-dependent products in high-income countries is stimulating cropland expansion in low-income countries.

Public engagement in pollinator initiatives tends to focus on raising awareness about pollinators and enabling people to act by describing simple actions that individual people can take. While this can motivate small-scale actions, and networks of actions at larger spatial scales [110], it can also stimulate inappropriate actions, and over-simplify complex socio-ecological issues. In addition, focusing solely on individual and local-scale action can allow larger actors (corporates, governments etc.) to downplay their own responsibility for action. Genuine and meaningful engagement with the complexities associated with restoring pollinator diversity at spatial and temporal scales, and in the associated potential wider and co-benefits and trade-offs, is required. Involvement of informed local stakeholders can enable better understanding of challenges and opportunities associated with pollinator conservation action. To this end, knowledge co-production in the design of conservation actions at local scale can help to ensure not only more effective action, but also social justice, leverage connections between policy and practice, and integrate pollinator action into wider sustainability and political issues [89].

### (e) Land management

What is actually done on the ground, how land is managed, is key to successfully restoring pollinator health. Our framework illustrates that the anthropogenic assets required to implement action on the ground are influenced by institutions, which are in turn a product of good quality of life. Thus, ultimately, transformative change in land management will only occur when there are system-level changes, incorporating the other elements of pollinator strategies outlined above (research and monitoring, education, and regulation).

It is possible to incorporate elements of transformative change into land management plans or strategies in pollinator initiatives. One interpretation of transformative change is that we need to ‘redesign’ agricultural systems, to make them highly productive but more resilient and sustainable in the long term [39]. Re-designed agricultural systems include integrated pest management, agroforestry, organic agriculture and various diversified farming systems. Hall & Steiner [23] demonstrated low or no inclusion of incentives for such farming systems in the US state pollinator-related legislature, but there is clearly potential to shape pollinator conservation actions around such systems.

## 5. Conclusion

In conclusion, viewing global pollinator conservation efforts through the lens of the IPBES conceptual framework reveals clear shortcomings in existing pollinator initiatives, which could be addressed by thinking more broadly about the relationship between people and nature. We highlight a number of areas where existing research can support efforts to move beyond these shortcomings. The challenge we see in pollinator conservation is not restricted to pollinators. It is replicated across all aspects of biodiversity conservation and climate change mitigation policy. Thus, through designing and implementing effective pollinator conservation actions, we can learn lessons that may be applied elsewhere. In addition, thinking about pollinator conservation in a broader context can help us to address the central issues of institutions, governance and indirect drivers and their role in reversing biodiversity loss.

## Data Availability

This article has no addtional data.
